# Assessing Nurses' Knowledge Regarding the Application of Artificial Intelligence Among Nursing Practice

**DOI:** 10.1155/nrp/9371969

**Published:** 2025-01-17

**Authors:** Mai M. Yaseen, Fatmah H. Alsharif, Reem A. Altaf, Taif W. Asiri, Rifan M. Bagies, Salwa B. Alharbi, Bothinah A. Altaf

**Affiliations:** ^1^Department of Public Health Nursing, Faculty of Nursing, King Abdulaziz University, Jeddah 21589, Saudi Arabia; ^2^Department of Medical Surgical Nursing, Faculty of Nursing, King Abdulaziz University, Jeddah 21589, Saudi Arabia; ^3^Department of Public Health Nursing, College of Nursing, King Abdulaziz University, Jeddah 21589, Saudi Arabia; ^4^Department of Statistics, Faculty of Science, King Abdulaziz University, Jeddah 21589, Saudi Arabia

## Abstract

Artificial intelligence (AI) is constantly improving the quality of medical procedures. Despite the application of AI in the healthcare industry, there are conflicting opinions among professionals, and limited research on its practical application in Saudi Arabia was conducted. Aim: To assess the nurses' knowledge regarding the application of AI in practice at one of the Ministry of Health hospitals in Saudi Arabia. Methods: Descriptive cross-sectional research using convenience sampling in January 2023 involving 307 staff nurses, using a single 11-item questionnaire. In addition, 6 closed-ended questions were used to assess the knowledge, possible risks, and advantages of AI. Results: All 307 participants completed the survey and used it for data analysis using SPSS V.25. Kruskal–Wallis and Whitney tests and descriptive statistics were used to identify the significant differences among groups. The study results reveal significant differences between age groups and working locations regarding familiarity with AI and future use of AI. In contrast, a considerable difference exists between licensed years groups regarding familiarity with AI. Surprisingly, education level does not affect AI knowledge. Additionally, the future use of AI is significantly affected by the nurse's gender. Limitation: Nurses were not included in previous studies on AI, and most nursing participants need more interest in AI. Conclusion: The study's results showed that nurses have positive opinions of AI in the healthcare industry, which will help them speed up procedures and reduce medical errors. AI applications can expand in healthcare by increasing the use of AI in the healthcare industry to improve care quality and encourage academic institutions to develop best practices for deploying AI applications in the healthcare industry.

## 1. Introduction

Alan Turing presented the concept of using computers to simulate intelligent behavior and critical thinking in 1950 [1]. Artificial intelligence (AI) is the research and creation of software applications that can carry out tasks that usually necessitate human intelligence, such as speech recognition, judgment, and language processing [2]. Moreover, all healthcare practitioners will be impacted by the revolutionary technology known as AI [3]. Furthermore, an AI system can aid in reducing unavoidable procedure errors in human nursing medicine [4]. Research emphasizing potential developments of AI in nursing practice has expanded in recent years [5]. Also, implementing AI and machine learning can enhance interprofessional collaboration, prevent accidents, improve nursing management, and improve nursing performance. It can also promote future global collaboration [6]. Nursing, throughout all fields of expertise, including management, therapeutic interventions, professional training, mentoring, regulation, and scientific studies, is anticipated to change due to AI [7].

As a result, aspiring nurses must be familiar with any of these innovations to administer comprehensive care in line with progressions in healthcare [8]. For instance, voice recognition technology can improve and help accelerate the paperwork of nursing practice [9]. Computer science has been employed to create a tool that assists the nursing staff in utilizing formalized techniques by instantly recommending the most appropriate phrases to use according to the text that the nurse has recorded [10]. To determine the answer to the proposed research question, “What is the nurse's understanding related to the application of AI technologies in nursing practice in Saudi Arabia?” A literature review was conducted using PubMed and Google Scholar.

The search used the following keywords: caregivers, healthcare workers, healthcare employees, medical field, perception, awareness, acceptations, and attitude. The study included research that was only conducted within the last 5 years. Since AI is now an emerging technology in the medical industry, limited research has been conducted in Saudi Arabia regarding the usage of AI, especially among nurses, necessitating continuous renewal of knowledge, particularly in healthcare.

Numerous research studies worldwide have concentrated on the knowledge of nurses and other healthcare professionals on implementing AI in practice. Six relative papers from Egypt, the United Arab Emirates, Turkey, and London, and one from Saudi Arabia, were included in this study. However, just a few studies have been conducted in Saudi Arabia to measure nurses' awareness of using AI in the field.

Synthesis of the literature indicated that the majority of nurse managers appeared to have a moderate understanding of using AI in nursing settings, and most of them appeared to have an optimistic response toward using AI in nursing settings. This indicates the significant positive relationship between nurse managers' jobs, education, and workplace demographic characteristics and their perception of using AI. Most of the nursing professionals who participated were familiar with AI and robot nurses. While most staff nurses consented that AI and robot nurses will not be capable of substituting for nurses, they would benefit nurses by reducing their workload [11, 12].

Even though innovation might alter how registered nurses utilize their time to deliver quality care, nurses will continue to be required. The nurses will discover new approaches to thinking and processing information; they will become data integrators, health instructors, and providers of patient services, assisted by technologies such as AI rather than being replaced by them [13].

According to Abuzaid, Elshami, and Fadden [14]; there is an inadequate understanding of AI concepts within a nursing career. Furthermore, education and training are essential to ensure AI's safe and smooth adoption into a nursing career. Nurses should enhance their understanding of basic AI and its use in nursing fields. The poll findings show a widespread lack of understanding of AI and awareness of its uses. Half of those polled had no idea what machine and deep learning were, and just 13% grasped the difference between the two. It was concluded that nurses need to continue educating and teaching about the AI research gap.

AI in healthcare informatics provides many opportunities that could completely transform the healthcare industry. Opportunities include improved diagnostics, customized treatment plans, clinical decision support systems (CDSS), predictive analytics for disease prevention, effective administrative procedures, telemedicine and remote patient monitoring, drug discovery and development, natural language processing (NLP), patient engagement and education, quality improvement and error reduction, research and insights, and patient engagement and education are some of the significant opportunities [15].

Abuzaid, Elshami, and Fadden [14] observed that even though using AI applications in nursing could assist nurses in making clinical decisions, there are some considerations that need to be addressed. First, AI education is insufficient in undergraduate nursing programs and ongoing education. Clinical information systems, data quality, standardization, data mining, and data analytics are just a few of the crucial educational components that should be included. AI technologies may facilitate responsive and evidence-based nursing practice in the nursing management system, for instance, by visualizing patient trends and offering insights for both short-term patient care and long-term planning and management. In order to support practice, nurses should also interact with the most relevant research and promote current understanding of the evidence. Conversely, numerous challenges face the application of AI in healthcare, including concerns about privacy and security of data, ethical issues, interoperability and integration, algorithmic bias, a lack of standards and regulations, transparency and trust, integration into clinical workflow, resource limitations, resistance to change, bias and quality of data in datasets, adherence to regulations, continuous learning and adaptation, and patient acceptance and engagement. Data security and privacy are crucial for avoiding unauthorized access or breaches, even though ethical considerations also include accountability, transparency, and potential bias in decision-making. Interoperability and integration across diverse healthcare platforms remain challenging due to using several standards and formats for data storage.

Castagno and Khalifa [16] looked into how AI concepts are implemented in nursing. However, there is little scientific proof in the currently available publications about the effectiveness of applying numerous artificially intelligent medical technologies in the medical industry. Nurses use fundamental nursing skills like assessment, planning, and outcome evaluation to give their patients optimal care. Nevertheless, not many nursing staffs are familiar with AI technologies. Although AI already affects the healthcare sector, little is acknowledged about how it could improve nursing services. Hence, the nursing staff shows limited awareness of AI concepts. Therefore, additional education and training are necessary to enable a smooth integration of AI into the nursing profession. Throughout the search process, only a few articles were conducted in Saudi Arabia, especially regarding the nursing profession and their knowledge regarding the application of AI in practice and the impact that will affect their outcome.

• Statement of the problem: AI is now an emerging approach in the health sector, and since limited research was conducted in Saudi Arabia, knowledge must continuously be updated, especially in the nursing industry. Therefore, measuring existing AI knowledge among nurses is crucial for identifying future training needs since they engage with patients using the technology.
• Purpose of the study: Assess nurses' knowledge regarding AI in Saudi Arabia. Additionally, it aims to ascertain the nurse's readiness, awareness, perception, and desire to integrate AI into nursing. Furthermore, to evaluate the potential contribution of AI applications to the nursing sector.
• Research question: What is the nurses' level of knowledge about the application and desire to implement AI technologies in Saudi nursing practice?

## 2. Materials and Methods

• Study design: This study utilized a quantitative, cross-sectional, descriptive research approach. A descriptive approach emphasizes nursing knowledge regarding AI utilization. A cross-sectional study was performed to gain information from data collected over a period of 6 months.
• Study setting: Saudi Arabia's KAU Faculty of Nursing in Jeddah is affiliated with this research project. Data from one of the Ministry of Health hospitals' nursing staff was collected in person using an online survey developed using Google Forms. The participants were selected from numerous departments at a Ministry of Health hospital in Jeddah, Saudi Arabia.
• Sampling and sample size: The data were collected via convenience sampling. It is crucial to recognize the limitations of convenience sampling, such as potential biases and reduced generalizability. In addition, the use of a large sample size reduces the impact of outliers, leading to more reliable data. Demonstrating that convenience sampling was common and accepted in similar research can justify its use, especially when resources, time, and accessibility are key constraints [17].
  The overall population is 1200 nurses, and the calculated sample size is 292, as determined by the sample size *G* power, to be confirmed with a margin of error of 5% and a confidence level of 95%. The entire sample was 307 participants, composed of female and male nursing employees who speak and read English and work at one of the Ministry of Health hospitals in Saudi Arabia. This excludes nursing students/interns and non-nursing healthcare employees.
• Data Collection Procedure: This research used a questionnaire to assess the perception of the knowledge and use of AI in healthcare practices.

### 2.1. Instrument

The tool consists of 11 items that divided into two parts: first the demographic information, namely, age, gender, nationality, educational attainment, and years of employment, were collected and included as shown in Table 1. The second part was the Attitudes toward Medical application of AI questionnaires developed by Oh et al. [18]. This part includes five questions using a 5-Likert scale that describes the attitude toward AI, the knowledge, and the use of AI in healthcare practices as shown in Table 2. Respondents rate each item on a five-point Likert scale ranging from 1 (strongly disagree) to 5 (strongly agree). The nursing knowledge regarding AI ranges from 5 to 25, where 5 indicate low knowledge, and 25 indicates high knowledge regarding AI [18].

In addition, six multiple-choice questions were included to collect advanced information concerning the application of AI such as advantages and disadvantages and fields of application of AI in healthcare fields. A total of two closed-ended questions (Q12 and Q13) about the advantages and disadvantages of AI in medical field; followed by two closed-ended questions (Q14 and Q15) on expected application. This was followed by two closed-ended questions (Q16 and Q17) on possible risks as participants were asked which problems they were concerned about regarding the application of AI in the healthcare field. The questions and possible answer choices are detailed in Table 3.

The validity of the questionnaire was originally conducted by Oh et al. [18]; who performed two validation steps. The first one involved a review of the instrument by the study researchers and an external panel of physicians, who indicated that the instrument was understandable and relevant. In the second step, the researcher conducted a pilot test on an external sample of 20 medical students and 80 physicians to ensure that the questionnaire was clear and reliable before the main study. Since the instrument was adopted with no change or translation of any part, it can be considered a valid instrument for our research.

The questionnaire's reliability was measured by Cronbach's Alpha *α* = 0.794, indicating that the questionnaire is reliable since Cronbach's Alpha > 0.75.

### 2.2. Recruitment Process

Most participants were nurses from outpatient and inpatient wards, who met the inclusion criteria, approached by researchers. Nurses were asked to participate in the study and gave them enough time to decide whether to join or not. Researchers introduced themselves and explained the study's objectives and the nature of the questions in details. Then, they received the survey form to complete it. The survey took between 3 to 5 min to fill out. Throughout the entire data collection and analysis process, confidentiality was maintained.

### 2.3. Ethical Consideration

The ethical approval of the Faculty of Nursing at King Abdulaziz University in Jeddah was obtained with the IRB number NREC Serial No: Ref No 1F. 16. The ethics committees at one of the Ministry of Health hospitals in Saudi Arabia granted permission to conduct the research. The permission to use the instrument was granted as it was published under open access terms and conditions as stated in the article copyrights which only required a proper citation within the current work. There was minimal risk to personal information; however, to avoid this risk, researchers ensured that the surveys did not have any personal information linked up to the participants and that all surveys were placed in a secure place for confidentiality. The decision to proceed with completing the survey constituted consent.

### 2.4. Analysis

SPSS version 25 was utilized to enter and analyze basic statistics, such as mean and standard deviation, which were used to describe the data. In the subgroup analysis, Kruskal–Wallis tests served to evaluate the effect of age, working location, education, and licensed years factors on questionnaire items. The differences in the questionnaire responses according to gender and nationality were analyzed using the Mann–Whitney test. For all tests, the level of significance was set at *p* ≤ 0.05.

## 3. Results and Discussion

In this study, 307 participants completed the questionnaire, representing a 95.9% rate of return. Table 1 shows the demographic characteristics of participants surveyed about AI. According to the research's findings, the majority of the participants were Saudi women aged 28 to 33 and of the female gender. The majority of them works in the medical field, has a bachelor's degree, and has been employed for seven or more years.

### 3.1. Response to Questionnaire

**Figure 1:**
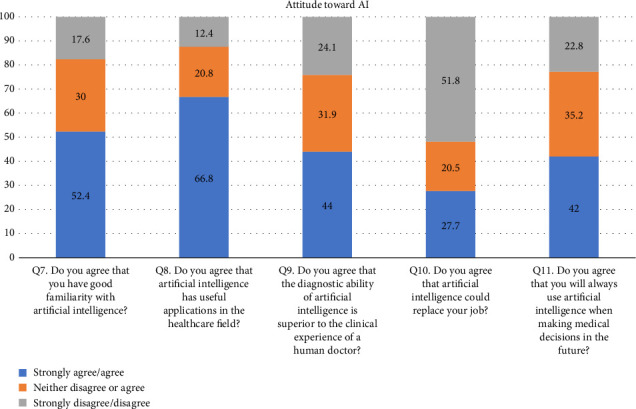
Q7–Q11. Attitudes toward artificial intelligence.

**Figure 2:**
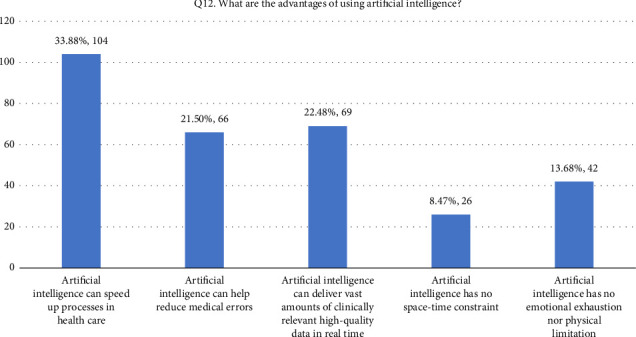
Q12. Advantages of artificial intelligence.

**Figure 3:**
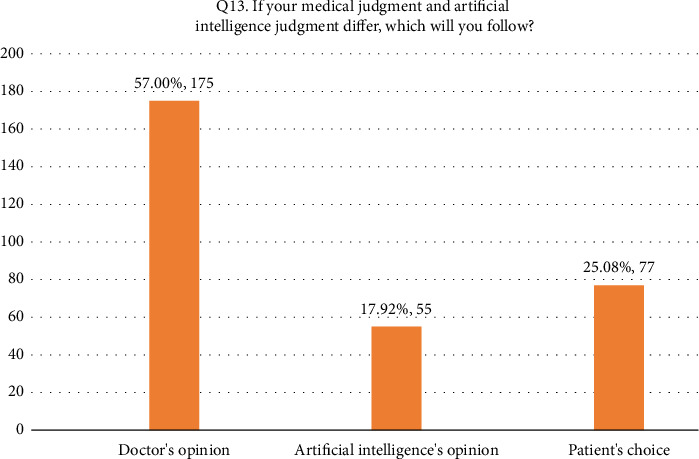
Q13. If medical judgment and AI judgment differ.

**Figure 4:**
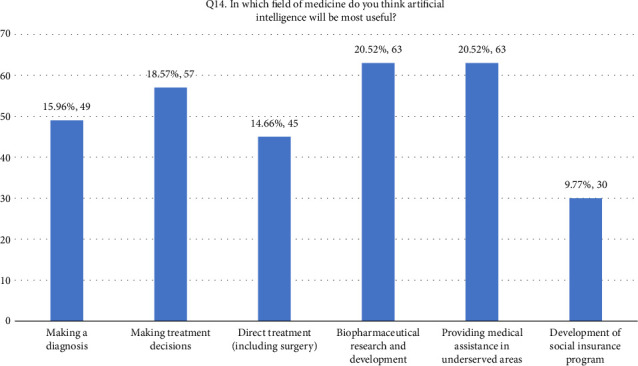
Q14. Application in medicine.

**Figure 5:**
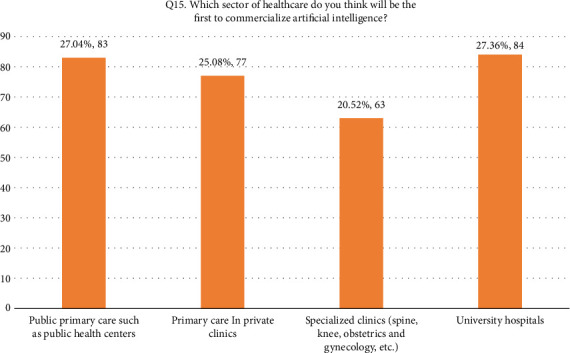
Q15. Application in healthcare.

**Figure 6:**
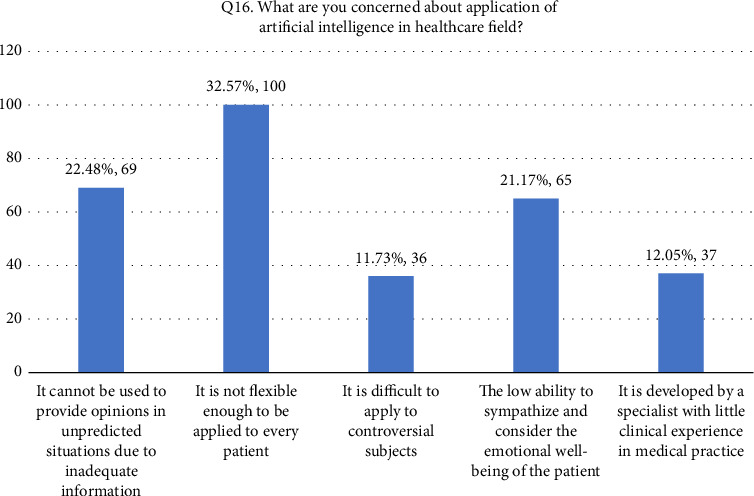
Q16. Possible risks-concerns about application of AI.

**Figure 7:**
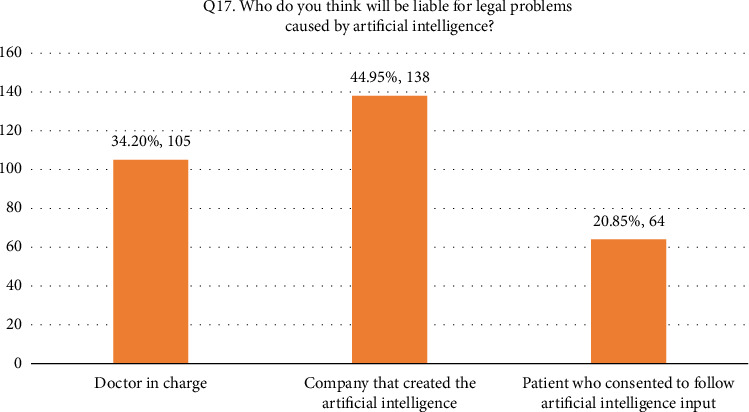
Q17. Possible risks-legal problems caused by AI.

• Attitude. According to this research, general familiarity with AI was acceptable since 52.4% of the participants responded that they were familiar with the term. In addition, 66.8% (205/307) of the participants considered AI useful in healthcare. Forty-four percent of the respondents agreed that AI's diagnostic ability is superior to a human doctor's clinical experience, whereas 24.1% of respondents did not support it. More than half of the respondents disagreed that AI could replace them. On the other hand, 27.7% agreed that AI would replace their job. Forty-two percent of the respondents agreed they would use AI in future tasks. The majority of the respondent's scores range between 13 and 21, indicating an average level of knowledge regarding the application of AI in nursing practice as shown in Figure 1.
• Advantages of AI. According to the results, 33.9% of the participants responded that AI can speed up processes in healthcare as shown in Figure 2. In addition, 22.5% of participants responded that AI can deliver vast amount of clinically relevant quality data in real-time as well as 21.5% responded that AI can help reduce medical errors. Moreover, 57% of participants responded that they will follow doctor's opinion when medical judgment and AI judgments differ as shown in Figure 3.
• Expected application in medicine. In this study, the respondents agreed that healthcare AI would be most useful in the future, including biopharmaceutical research and development and providing medical assistance in underserved areas (20.5%). Less than 10% felt that AI would be useful in developing social insurance programs. 16%–18% of the participants expected AI to be used in making diagnosis and treatment decisions, respectively. In comparison, 14% of them expected AI to be useful in direct treatment (including surgery), as shown in Table 2. The respondents felt that university hospitals and public primary care are expected to be the first sectors to commercialize AI (27%, 27.4%). Comparatively, private and specialized clinics come after 25.1% and 20.5%, respectively, as shown in Figures 4 and 5.
• Possible risks. According to the participants, the possible issue concerning the application of AI in the Healthcare field is that AI is not flexible enough to be applied to every patient (32.6%). In addition, they felt that AI would fail to provide opinions in unpredicted situations 22.5%. Also, the lack of emotional well-being would be another issue with the application of AI (21.6%). 12.1%–11.7% of the participants were concerned that AI was developed by a specialist with little clinical experience in medical practice and the difficulties of applying AI to controversial subjects, respectively, as shown in Figures 6 and 7.

### 3.2. Subgroup Analysis

Table 4 shows the subgroup analysis of participant attitudes toward AI according to age, working location, education level, and license years. The analysis shows significant differences between groups in Q7 according to work location and year licensed, Q8 according to age, Q10 according to age and education, and Q11 according to age. In contrast, there are no significant differences between groups in Q9 according to all factors, Q7 according to age and education, Q8 according to education and licensed years, Q10 according to licensed years, and also Q11 according to work location, education, and year licensed. Post hoc tests were used to conduct pairwise comparisons.

### 3.3. Familiarity With AI (Q7)

Although the Kruskal–Wallis shows significant differences between groups in Q7 according to work location and year licensed, the pairwise comparisons show that there is only a substantial difference in Q7 according to licensed years (between groups (22–27) and (46 and above). On the other hand, the comparisons show no significant difference between groups according to work (*p* value = 0.043), which is very close to the significant level (*α* = 0.05).

### 3.4. Comparisons by Education Level

The analysis shows an insignificant difference in Q7-Q8-Q9-Q11 according to education level (*α* = 0.05). However, only Q10 differs significantly between education levels (Master's, Diploma, and Bachelor's).

### 3.5. Comparisons by Age

The analysis shows a significant difference in Q8 and Q10 according to age. These differences in Q8 are significant between age groups (22–27 group and 46 and above group). The differences in Q10 are significant between age groups (22–27 and 28–33 groups).

### 3.6. Comparisons by Licensed Years

The analysis shows a significant difference in Q8 according to licensed years. These differences are significant between participants who are (one to three licensed years and less than 1 year). Also, the differences between the groups (one to three licensed years and 4–6 years) are significant.

### 3.7. Comparisons by Work Location

The analysis shows significant differences in Q8 and Q10 according to working location at (*α* = 0.05). These differences are significant in Q8 between groups OR and (ICU-Medical), respectively, as the pairwise comparisons show (*p* value = 0.013 and 0.047). On the other hand, the pairwise comparisons show a significant difference in Q10 between ER and medical groups (*p* value = 0.016).

### 3.8. Comparison by Gender

Mann–Whitney is used to evaluate participant attitudes toward AI according to gender as shown in Table 5. The results show a significant difference in Q10 according to gender (*p* value = 0.042). In contrast, there is no significant difference according to gender in Q7, Q8, Q9, and Q11.

### 3.9. Comparison by Nationality

Mann–Whitney is used to evaluate participant attitudes toward AI according to gender as shown in Table 5. The results show a significant difference in Q10 and Q11 according to nationality (*p* value = 0.034, 0.042). In contrast, there is no significant difference according to gender in Q7, Q8, and Q9.

## 4. Discussion

In this study, 307 participants out of 320 completed the questionnaire, representing a 95.9% rate of return. A major development in healthcare informatics, AI improves patient care, treatment planning, diagnosis, and administrative tasks. Applications for it include pathology, CDSS, medical imaging, NLP, predictive analytics, virtual health assistants, drug research, remote patient monitoring, fraud detection, and security. Furthermore, ethical considerations are integrated into the application of AI [15]. In this study, researchers questioned the participants about whether AI had better diagnostic abilities than human doctors. Less than half of the participants concurred that AI would be better at diagnosis. Consistent with the literature [19], assert that AI may be better than a doctor's clinical judgment and can aid physicians in selecting the best assessment and treatment plan for a specific disease. Reference [20], also mentioned that the latest AI advancement provides diagnosis capabilities equivalent to physicians, particularly in image recognition-related areas.

Moreover, it was discovered that a large number of Saudi nurses do not believe AI will take over their jobs after receiving responses from them. Similarly, most researchers concur that nurses will not be directly replaced by AI soon. Consistent with the literature conducted by [21], AI ought to enhance, not take over, nursing care's particular abilities and emotional qualities. However, new nurse-patient communications incorporating AI are anticipated in clinical practice, which could improve nurse-patient relationships and provide safe, high-quality care [7]. Nurses recruited in this study are most worried that AI is not adaptable enough to be used with every patient. Similarly, doctors, as evidenced in the literature, seem to be well cognizant that AI is not sufficiently flexible to accommodate all the requirements and needs of the patients they see on a regular basis [22].

In addition, if the doctors' and AI's judgments differed, most participants declared that they would follow the doctor's instructions. This finding aligns with previous research on human-AI interactions indicating that individuals prefer human doctors over AI-made decisions [23]. It was assumed that nurses' responses regarding following the AI judgment would be limited because the AI is not being employed effectively in the scope of nursing interventions, as observed throughout the data collection in the hospital.

According to the findings of this study, nurses have a significant level of understanding of AI. Many of the participants assumed AI could be useful in the medical industry. Nurses believed that AI would be most beneficial for speeding up procedures in healthcare. Consistent with the literature of [24], AI was rapidly improving healthcare services and making the healthcare workload easier. Moreover, Kocakoç [25] mentioned that medical institutions will evolve into a framework supported by AI, which enhances and streamlines healthcare processes. This system will also offer patients access to essential services and information while minimizing dependence on medical personnel. Less than half of the respondents agreed that AI could be superior to a doctor's clinical experience. However, this review highlights the shortcomings of clinical decision-support systems used by doctors and proposes that AI can assist physicians in making improved medical decisions. Automated AI models that utilize data from real-time electronic health records and mobile device outputs have the potential to address these challenges [26]. The majority of Saudi nurses do not believe that AI will replace their jobs. If the doctors' and AI's judgments differ, most respondents agreed to follow the doctor's opinion. Furthermore, many nurses are concerned that AI is not flexible enough to be applied to every patient.

In terms of ethical implications, it was really important to highlight the ethical part, as multiple studies believe that AI has the potential to transform healthcare completely. A successful AI-driven healthcare system must take into account and address a number of important factors, including informed consent, high standards of safety and efficacy, cyber resilience and cybersecurity, high levels of data protection and privacy, algorithmic fairness, an adequate level of transparency and regulatory oversight, and an ideal liability regime for AI [27]. To reach the desired goal that AI benefits everyone, we need to build a system based on public trust. Patients should feel comfortable knowing that their data is being handled responsibly; AI systems must be clear and understandable in order to encourage accountability and redress. AI systems should be utilized to improve human contact and empathy, not to replace it. Informed permission is essential. AI holds great promise for enhancing our healthcare system; yet, in order to maximize this potential, we must begin addressing the ethical and legal issues at hand right away [28].

### 4.1. Limitations

This study had a few limitations. One of the limitations of this study was the convenience sampling used in this research, which could lead to potential bias and generalizability. Another limitation of this study was that the participants did not have a sufficient background in AI, which could lead to potential bias, while researchers provided examples of AI to assist them in understanding some of the survey questions. Additionally, all participants were recruited from one hospital; therefore, the results cannot be generalized to other hospital and nursing populations.

### 4.2. Recommendations for Future Study

In order to increase the realizability of findings, a larger sample size from different hospitals would be needed to have a more representative sample. Future studies should contain more diversity in the demographic data of participants, especially those who have a background in AI, by targeting hospitals that use AI in nursing practice. Moreover, a qualitative study design would explore nurses' perceptions and backgrounds using observation interviews and/or focus groups, adding more in-depth details to the content. Most importantly, similar research should be conducted in Saudi Arabia with a focus on nursing care-specific and guiding policy or training programs in nursing.

## 5. Conclusion

According to this study's results, nurses believed that AI technology is being used in collaboration with nursing to speedily synthesize information, finish tasks, aid with clinical decisions, and enhance patient outcomes. Most of the research participants have a positive view of AI, and nurses have a significant level of understanding of AI. According to the findings of this study, nursing staff believe that implementing AI in healthcare might be beneficial in the medical industry, and a large number of Saudi nurses do not believe AI will take over their jobs. Although most nurses were worried that AI is not adaptable enough to be used with every patient, nurse practitioner suspected that AI would be most effective in speeding up procedures and reducing medical errors in healthcare. Additionally, less than half of the respondents agreed that AI could be superior to a doctor's clinical experience. However, If the doctors' and AI's judgments differ, most respondents agreed to follow the doctor's opinion. Finally, half of the nurses did not believe AI might replace their position as healthcare professionals.

### 5.1. Implication to Nursing

Encourage the development of essential AI applications and expansion in healthcare by increasing the use of AI in the healthcare industry to improve care quality and encourage academic institutions to participate in developing and endorsing best practices for deploying AI applications in the healthcare industry.

## Figures and Tables

**Table 1:** Demographic characteristics.

Characteristic	*n* (%)
*Age (years)*	
22–27	74 (24.1)
28–33	107 (34.9)
34–39	74 (24.1)
40–45	30 (9.8)
46 and above	22 (7.2)

*Gender*	
Female	260 (84.7)
Male	47 (15.3)

*Nationality*	
Saudi	207 (67.4)
Non-Saudi	100 (32.6)

*Education*	
Diploma	77 (25.1)
Bachelor degree	201 (65.5)
Master degree	18 (5.9)
Ph.D. degree	11 (3.6)

*Area*	
Medical	99 (32.2)
Surgical	50 (16.3)
OR	23 (7.5)
ER	28 (9.1)
ICU	17 (5.5)
Others	90 (29.3)

*Licensed years*	
Less than 1 year	49 (16)
1–3 years	42 (13.7)
4–6 years	47 (15.3)
7 years or more	169 (55)

**Table 2:** Participants' attitudes on artificial intelligence (*N* = 307).

Question	*n* (%)
Attitudes	
*Q7. Do you agree that you have good familiarity with artificial intelligence?*	
Strongly agree/agree	161 (52.4)
Neither disagree or agree	92 (30)
Strongly disagree/disagree	54 (17.6)

*Q8. Do you agree that artificial intelligence has useful applications in the healthcare field?*	
Strongly agree/agree	205 (66.8)
Neither disagree or agree	64 (20.8)
Strongly disagree/disagree	38 (12.4)

*Q9. Do you agree that the diagnostic ability of artificial intelligence is superior to the clinical experience of a human doctor?*	
Strongly agree/agree	135 (44)
Neither disagree or agree	98 (31.9)
Strongly disagree/disagree	74 (24.1)

*Q10. Do you agree that artificial intelligence could replace your job?*	
Strongly agree/agree	85 (27.7)
Neither disagree or agree	63 (20.5)
Strongly disagree/disagree	159 (51.8)

*Q11. Do you agree that you will always use artificial intelligence when making medical decisions in the future?*	
Strongly agree/agree	129 (42)
Neither disagree or agree	108 (35.2)
Strongly disagree/disagree	70 (22.8)

**Table 3:** Advanced information.

Question	*n* (%)
*Q12. What are the advantages of using artificial intelligence?*	
Artificial intelligence can speed up processes in healthcare	104 (33.9)
Artificial intelligence can help reduce medical errors	66 (21.5)
Artificial intelligence can deliver vast amounts of clinically relevant quality data in real-time	69 (22.5)
Artificial intelligence has no space-time constraint	26 (8.5)
Artificial intelligence has no emotional exhaustion nor physical limitation	42 (13.7)

*Q13. If your medical judgment and artificial intelligence judgments differ, which will you follow?*	
Doctor's opinion	175 (57)
Artificial intelligence's opinion	55 (17.9)
Patient's choice	77 (25.1)

*Expected fields*	

*Q14. Which field of medicine do you think artificial intelligence will be most useful?*	
Making a diagnosis	49 (16)
Making treatment decisions	57 (18.6)
Direct treatment (including surgery)	45 (14.7)
Biopharmaceutical research and development	63 (20.5)
Providing medical assistance in underserved areas	63 (20.5)
Development of social insurance program	30 (9.8)

*Q15. Which healthcare sector do you think will be the first to commercialize artificial intelligence?*	
Public primary care, such as public health centers	83 (27)
Primary care in private clinics	77 (25.1)
Specialized clinics (spine, knee, obstetrics, and gynecology, etc.)	63 (20.5)
University hospitals	84 (27.4)

*Possible risk*	

*Q16. What are you concerned about the application of artificial intelligence in the healthcare field?*	
It cannot be used to provide opinions in unpredicted situations due to inadequate information	69 (22.5)
It is not flexible enough to be applied to every patient	100 (32.6)
It is difficult to apply to controversial subjects	36 (11.7)
The low ability to sympathize and consider the emotional well-being of the patient	65 (21.2)
It is developed by a specialist with little clinical experience in medical practice	37 (12.1)

*Q17. Who do you think will be liable for legal problems caused by artificial intelligence?*	
Doctor in charge	105 (34.2)
Company that created the artificial intelligence	138 (45)
Patient who consented to follow artificial Intelligence's input	64 (20.8)

**Table 4:** Comparisons between groups according to age, working location, education attainments, and licensed years (Kruskal–Wallis test).

Attitude					
Questions		Age	Working location	Education	Licensed years
Q7	Do you agree that you have good familiarity with artificial intelligence?	0.391	0.043⁣^∗^	0.310	0.013⁣^∗^
Q8	Do you agree that artificial intelligence has useful applications in the healthcare field?	0.005⁣^∗^	0.012⁣^∗^	0.751	0.080
Q9	Do you agree that the diagnostic ability of artificial intelligence is superior to the clinical experience of a human doctor?	0.496	0.090	0.090	0.803
Q10	Do you agree that artificial intelligence could replace your job?	0.002⁣^∗^	0.018⁣^∗^	0.001⁣^∗^	0.110
Q11	Do you agree that you will always use artificial intelligence when making medical decisions in the future?	0.046⁣^∗^	0.186	0.913	0.416

⁣^∗^Indicate significant differences between groups.

**Table 5:** Comparisons according to gender and nationality (Mann–Whitney test).

Questions		Nationality	Gender
Q7	Do you agree that you have good familiarity with artificial intelligence?	0.608	0.443
Q8	Do you agree that artificial intelligence has useful applications in the healthcare field?	0.211	0.069
Q9	Do you agree that the diagnostic ability of artificial intelligence is superior to the clinical experience of a human doctor?	0.223	0.924
Q10	Do you agree that artificial intelligence could replace your job?	0.034⁣^∗^	0.42⁣^∗^
Q11	Do you agree that you will always use artificial intelligence when making medical decisions in the future?	0.042⁣^∗^	0.93

^∗^Significance of value *p* = 0.000 < 0.05.

## Data Availability

Data available on request, by contacting the corresponding author Mai M. Yaseen at myasin@kau.edu.sa.
